# Uncontrolled Hypertension in Cardio‐Renal‐Metabolic Diseases: Real‐World Insights on Patient Profiles and Management Patterns From iCaReMe Global Registry

**DOI:** 10.1155/ijhy/9668123

**Published:** 2026-09-03

**Authors:** Kamlesh Khunti, Mustafa Arici, Carol Pollock, Chien-Ning Huang, Rubén Silva-Tinoco, Adel A. El Sayed, Esteban Coto, Karim Salama, Hardik Vasnawala, Ahmed Hadaoui

**Affiliations:** ^1^ Diabetes Research Centre, University of Leicester, Leicester, UK, le.ac.uk; ^2^ Department of Nephrology, Hacettepe University Faculty of Medicine, Ankara, Türkiye, hacettepe.edu.tr; ^3^ Kolling Institute, Royal North Shore Hospital, University of Sydney, Sydney, Australia, sydney.edu.au; ^4^ School of Medicine, Chung Shan Medical University, Taichung, Taiwan, csmu.edu.tw; ^5^ Clinic Specialized in Diabetes Management, Servicios Públicos de Salud del Instituto Mexicano del Seguro Social para el Bienestar, Mexico City, Mexico; ^6^ Internal Medicine, Sohag Faculty of Medicine, Sohag, Egypt; ^7^ Global Medical Affairs, BioPharmaceuticals Medical, AstraZeneca, Gaithersburg, Maryland, USA, astrazeneca.com; ^8^ Medical Affairs, AstraZeneca International, Dubai, UAE; ^9^ Medical Affairs, AstraZeneca International, AstraZeneca Algeria, Algiers, Algeria

## Abstract

**Background:**

Hypertension (HTN) is a leading risk factor for cardiovascular (CV) and renal morbidity and mortality, yet blood pressure (BP) control remains suboptimal worldwide. Understanding management patterns, especially among patients with coexisting Type 2 diabetes mellitus (T2DM), chronic kidney diseases (CKDs), or heart failure (HF), and assessing uncontrolled HTN burden across diverse health systems are critical to inform guideline‐concordant strategies to improve care.

**Methods:**

Cross‐sectional analysis includes participants with HTN, with or without comorbid T2DM, CKD, or HF enrolled in iCaReMe Global Registry across 29 countries. Demographic and clinical characteristics, antihypertensive treatment patterns, prevalence of uncontrolled HTN (defined as SBP ≥ 130 mmHg or DBP ≥ 80 mmHg), and its associated factors were assessed.

**Results:**

Among 23,063 participants, comorbidities included T2DM (71.4%), CKD (38.2%), and HF (14.6%). Key CV risk factors were family history of CV disease (34.3%), obesity (40.5%), and dyslipidemia (51.0%). Echocardiography (available in 28.6%) revealed left ventricular hypertrophy (LVH) in 30.6% and diastolic dysfunction in 42.4%. Overall, 88.7% were prescribed antihypertensive drugs: renin–angiotensin system (RAS) blockers (76.6%), calcium channel blockers (CCBs, 42.0%), beta‐blockers (41.4%), diuretics (23.7%), with a majority (62.3%) on combination regimens. Uncontrolled HTN was prevalent in 75.4%, who were younger (59.8 vs 61.8 years) and exhibited higher burden of obesity (42.5% vs 34.6%), CKD (39.3% vs 33.1%), LVH (32.9% vs 24.8%), and treated less intensively with antihypertensives (60.2% vs 88.7%) and combinations (46.2% vs 61.8%) when compared to the controlled HTN group. Multivariate analysis confirmed independent association of non‐use of RAS blockers/CCB/diuretics combinations (dual/triple therapy), obesity, and comorbid CKD, with uncontrolled HTN (OR [95% CI], 2.8 [2.44–3.18]/2.3 [1.66–3.16], 1.5 [1.38–1.65], and 1.4 [1.27–1.53], respectively, *p* < 0.0001).

**Conclusion:**

This study uncovers alarming rates of uncontrolled HTN in real‐world settings, exacerbated by comorbid T2DM/CKD/HF, underscoring the need to identify HTN management gaps and tailor innovative strategies to mitigate CV risk.

**Trial Registration:** ClinicalTrials.gov identifier: NCT03549754

## 1. Introduction

Hypertension (HTN) is a global health challenge, affecting 1.4 billion individuals worldwide, with two‐thirds living in low‐income and middle‐income countries (LMIC) [1, 2]. This condition is recognized as the leading preventable risk factor for global mortality, contributing to approximately 10 million deaths annually [3]. In fact, this mortality burden is largely driven by HTN’s role as the primary risk factor for cardiovascular diseases (CVDs), accounting for 49% of coronary heart diseases, 62% of stroke cases, and a 71% increase of heart failure (HF) risk [4, 5]. Furthermore, HTN significantly increases the risk of chronic kidney diseases (CKDs) with a hazard ratio of 1.39–1.79 [6] and contributes to the progression of CKD [7, 8].

The clinical landscape of HTN is frequently complicated by the presence of coexisting conditions, notably Type 2 diabetes mellitus (T2DM), CKD, and HF. The relationship between HTN and these comorbidities is not just co‐occurrence but a complex interplay that dramatically amplifies adverse cardiovascular and renal outcomes [9–13].

The global burden of HTN has made it a central focus of numerous scientific societies worldwide, leading to the development of several guidelines over decades aiming to improve awareness, enhance diagnosis, optimize its control, and ultimately reduce its devasting impact on cardiovascular and renal health. Major guidelines from the World Health Organization (WHO) in 2021, the European Society of Hypertension (ESH) in 2023, the European Society of Cardiology (ESC) in 2024, and the American College of Cardiology/American Heart Association/(ACC/AHA) in 2025 have evolved to reflect the best available evidence for all aspects of HTN management [14–17]. These guidelines present similarities and differences, particularly concerning the definition of HTN, thresholds for treatment initiation, and specific blood pressure (BP) targets. While 2025 ACC/AHA guidelines notably lowered the definition of HTN to ≥ 130/80 mmHg and recommended a target of < 130/80 mmHg [17], the 2021 WHO [14], 2023 ESH [15], and 2024 ESC guidelines [16] maintain a definition of ≥ 140/90 mmHg. The WHO advocates a treatment target of < 140/90 mmHg in patients without comorbidities and a systolic BP of < 130 mmHg for those with established CVD [14], whereas ESH recommended a general target of < 140/80 mmHg, with a more intensive systolic target of 120–129 mmHg, if tolerated [15]. Furthermore, 2024 ESC guidelines introduced an “elevated BP” category and advocated for strict systolic BP targets of 120–129 mmHg for most patients including those with comorbidities like T2DM, CKD, and HF [16]. In spite of these differences in thresholds and targets, all guidelines consistently underscore the foundational role of nonpharmacological interventions, the importance of comprehensive CV risk assessment, and the frequent need for combination pharmacotherapy, often prioritizing renin–angiotensin system (RAS) blockers to achieve optimal BP control.

Despite the availability of evidence‐based guidelines, straightforward diagnostic methods, and cost‐effective treatments, the real‐world management of HTN remains inadequate. Globally, more than half of adult cases go undiagnosed and only 44% of adults with HTN receive treatment, while less than one in four achieve adequate BP control—all reflecting substantial disparities across countries [1, 2]. This gap is especially pronounced in low‐ and middle‐income countries (LMICs), where most patients with HTN live and just 1 in 10 patients have BP under control [18].

Efforts to mitigate the global HTN burden are hindered by significant gaps in real‐world evidence on disease management, particularly within LMICs. Traditional clinical trials often fail to reflect the broad diversity of patient populations, healthcare settings, and the unique challenges encountered beyond controlled environments. Real‐world data registries can help bridge these gaps by systematically capturing standardized information on patient risk factors, local treatment practices, medication adherence, and health outcomes, especially in resource‐limited settings, enabling stakeholders throughout the healthcare system to address key clinical, operational, and economic barriers, answer critical questions, and support delivery of high‐quality HTN care.

The ongoing iCaReMe Global Registry has been designed to provide comprehensive real‐world evidence on the management of HTN, T2DM, CKD, and HF. It is the extension of the global DISCOVERing Treatment Reality of Type 2 Diabetes in Real‐World Settings (DISCOVER) Registry and continues collecting data on patients’ characteristics, disease management patterns, healthcare resource utilization, and clinical outcomes across diverse global settings. It offers the opportunity to capture data from a broad spectrum of regions, healthcare systems, and patient demographics, thereby providing insights into the diverse patterns of disease presentation and management across the globe.

In this article, we report the characteristics and management patterns in participants with HTN enrolled in the iCaReMe Global Registry along with the burden and determinants of uncontrolled HTN.

## 2. Methods

### 2.1. Study Design and Participants

The iCaReMe Global Registry is a multinational, observational study assessing the quality of care and management of cardiovascular, renal, and metabolic (CVRM) diseases in real‐world settings. The registry enrolls adult patients with confirmed diagnosis of HTN, T2DM, CKD, and/or HF as determined by the treating investigator based on established local clinical practice guidelines and/or documented within the patient medical records. It involves noninterventional data collection from participants undergoing clinical assessments and receiving standard medical care as part of the routine clinical practice. The study is conducted in accordance with ethical principles outlined in the Declaration of Helsinki, International Council for Harmonization (ICH) Good Clinical Practice, Good Pharmacoepidemiology Practices and applicable local laws, regulations, and guidelines. iCaReMe Global Registry protocol was approved by ethics committees, institutional review boards of the participating sites, and written consent was obtained from every participant before study enrollment.

For this report, we conducted a cross‐sectional analysis of baseline data from participants with HTN enrolled in the iCaReMe Global Registry between February 2018 and October 2024 across 29 countries (Argentina, Brazil, Costa Rica, Ecuador, Egypt, Ethiopia, Georgia, Ghana, Greece, Hong Kong, India, Indonesia, Iraq, Jordan, Kazakhstan, Kenya, Lebanon, Malaysia, Mexico, Nigeria, Peru, the Philippines, Russia, South Africa, Taiwan, Thailand, Türkiye, Ukraine, United Arab Emirates). We included all patients with a documented diagnosis of HTN at enrollment, regardless of the presence of other comorbid conditions (T2DM, CKD, and/or HF). The study objectives were to (a) characterize the sociodemographic and clinical characteristics, (b) describe current management patterns and treatment approaches, and (c) assess the burden and clinical features associated with uncontrolled HTN (systolic BP [SBP] ≥ 130 mmHg or diastolic BP [DBP] ≥ 80 mmHg).

### 2.2. Study Data

In this study, baseline data of patients with HTN with or without comorbidities from the iCaReMe Global Registry were captured. Patient first visit was considered as baseline, and the following data were extracted: (a) sociodemographic characteristics (country, gender, age, smoking status, education level, working status, insurance); (b) medical history (personal and family history of CVD, relevant comorbidities, duration of disease); (c) clinical and physical examination (office SBP and DBP, body mass index [BMI], two‐dimensional [2D] echocardiography [ECHO], laboratory [Lab] test results such as glycated hemoglobin [HbA1c], lipid profile, serum creatinine, estimated glomerular filtration rate [eGFR] using the 2021 CKD epidemiology collaboration race‐free estimated glomerular filtration rate equation, urine albumin‐creatinine ratio [uACR]); (d) prescribed treatment (pharmacological class of antihypertensive drugs grouped as alpha blockers, angiotensin‐converting enzyme inhibitors [ACEi], angiotensin receptor blockers [ARBs], beta blockers, CCBs, central acting agents, diuretics [thiazide diuretics, thiazide‐like diuretics, loop diuretics, potassium sparing diuretics except mineralocorticoid receptor antagonists], mineralocorticoid receptor antagonists [MRA], renin inhibitors, and direct vasodilators).

### 2.3. Statistical Analysis

Patient social and demographics, medical history, clinical characteristics, and treatment patterns were summarized descriptively. Categorical variables were presented using frequencies, percentages, and continuous variables using the number of observations, arithmetic mean, and standard deviation (SD). For the comparison of subgroups, Student’s *t*‐test/ANOVA were used for continuous variables and Chi‐square/Fisher’s exact test for categorical variables. Unknown and missing data were excluded from percentage calculations.

Analysis was performed in the overall patient population and in subgroups stratified by

• Mutually exclusive comorbidity: defined by the presence of either T2DM alone, CKD alone, HF alone, or none of these comorbidities (HTN alone),
• Geographic region: Asia–Pacific (APAC; Hong Kong, India, Indonesia, Malaysia, the Philippines, Taiwan, and Thailand), Eurasia (Georgia, Greece, Kazakhstan, Russia, and Ukraine), Latin America (LATAM; Argentina, Brazil, Costa Rica, Ecuador, Mexico, and Peru), and Middle East and Africa (MEA; Egypt, Ethiopia, Ghana, Iraq, Jordan, Kenya, Lebanon, Nigeria, South Africa, Türkiye, United Arab Emirates),
• HTN control status: controlled HTN defined as SBP < 130 mmHg and DBP < 80 mmHg and uncontrolled HTN defined as SBP ≥ 130 mmHg or DBP ≥ 80 mmHg.

Determinants of uncontrolled HTN were identified using stepwise logistic regression; candidate covariates included sociodemographic, clinical, and treatment variables. Variables with *p* ≤ 0.1 in univariate analysis were entered into a multivariable logistic regression model. The magnitude of association between the covariates and uncontrolled HTN was measured using odds ratios (OR) with 95% confidence interval (CI); statistical significance was set at *p* < 0.05.

All analyses were performed using the SAS statistical software system, version 9.2 (SAS Institute, Inc., Cary, NC, USA).

## 3. Results

The study involved 23,063 participants with HTN from 29 countries across four geographic regions. Detailed sociodemographic and clinical characteristics, along with antihypertensive treatment patterns, are presented in Table 1.

**TABLE 1:** Sociodemographic, clinical characteristics, and treatment patterns of the study population.

Characteristics^#^	HTN overall (*N* = 23,063)
Age, *n*	23,027
Mean (SD), years	60.3 (12.04)
Female	11,966 (51.9)
Duration of HTN, *n*	17,090
Mean (SD), years	9.8 (8.63)
Ethnicity, *n*	12,579
Arabic	587 (4.7)
Asian—Chinese	564 (4.5)
Asian—East	93 (0.7)
Asian—Other	1115 (8.9)
Asian—South	673 (5.4)
Black	2762 (22.0)
Caucasian	2972 (23.6)
Hispanic	2226 (17.7)
Mixed (e.g., brown, mulatto, others)	1239 (9.8)
Native American	7 (0.1)
Others	341 (2.7)
Main Working Status, *n*	11,783
Employed	2850 (24.2)
Self‐Employed	2154 (18.3)
Retired	2415 (20.5)
Disabled	156 (1.3)
Not working	4208 (35.7)
Education Level, *n*	1,1840
No formal education	936 (7.9)
Primary (1–6 years of education)	2693 (22.7)
Secondary (7–13 years of education)	4381 (37.0)
University/Higher Education (greater than 13 years of education)	3830 (32.3)
Health Insurance Coverage, *n*	11,325
Mixed	274 (2.4)
No insurance	2608 (23.0)
Private	1641 (14.5)
Public/governmental	6802 (60.1)
Comorbidities	
History of T2DM	16,458 (71.4)
Mean (SD) HbA1c, %	7.9 (1.92)
History of CKD	8803 (38.2)
Mean (SD) eGFR, mL/min/1.73 m^2^	43.3 (29.94)
History of HF	3371 (14.6)
Mean (SD) LVEF, %	42.1 (13.66)
HTN without T2DM, CKD or HF	2566 (11.1)
Cardiovascular Risk Factors	
Age (≥ 65 years)	8755 (38.0)
Family History of CVD	5887 (34.3)
Current smoker	1659 (7.7)
Ex‐smoker	2664 (12.4)
Obesity (BMI ≥ 30 kg/m^2^)	7910 (40.5)
History of Dyslipidemia	9596 (51.0)
HbA1c, *n*	16,477
Mean (SD), %	7.5 (1.97)
LDL‐c, *n*	15,153
Mean (SD), mg/dL	96.6 (43.96)
Hypertension‐Mediated Organ Damage	
2D Echocardiography	6602 (28.6%)
LVH	2020 (30.6)
Diastolic dysfunction	2796 (42.4)
LAE	1805 (27.3)
eGFR (CKD‐EPI 2021) , *n*	16,474 (71.4)
eGFR 30–59 mL/min/1.73 m^2^	3955 (24.0)
uACR, *n*	8694 (37.7)
uACR 30–300 mg/g	2787 (32.1)
Established CVD and CKD	
History of coronary artery disease	3035 (18.0)
History of stroke	769 (4.4)
eGFR < 30 mL/min/1.73 m^2^	2947 (17.9)
uACR > 300 mg/g	1572 (18.1)
ECG, *n*	7475
Atrial fibrillation	513 (6.9)
Office Blood pressure Measurements	21,837 (94.7)
Mean (SD) SBP, mmHg	134.6 (19.91)
Mean (SD) DBP, mmHg	79.7 (12.46)
SBP ≥ 130 mmHg or DBP ≥ 80 mmHg	16,462 (75.4)
SBP ≥ 140 mmHg or DBP ≥ 90 mmHg	10,037 (46.0)
Treatment Patterns, *n*	21,654
Antihypertensive medications	19,205 (88.7)
ACEi/ARB^∗^	14,705 (76.6)
Alpha blockers	583 (3.0)
Beta blockers	7949 (41.4)
CCB	8059 (42.0)
Central acting agents	622 (3.2)
Diuretics^1^	4561 (23.7)
Vasodilators	452 (2.4)
MRA	1911 (10.0)
Renin‐Inhibitors	3 (0.0)
Antihypertensive regimens	
1 antihypertensive drug	7233 (37.7)
2 antihypertensive drugs	6343 (33.0)
ACEi/ARB^∗^ + CCB	2433 (38.4)
ACEi/ARB^∗^ + Diuretics^1^	758 (12.0)
3 antihypertensive drugs	3766 (19.6)
ACEi/ARB^∗^ + CCB + Diuretics^1^	662 (17.6)
≥ 4 antihypertensive drugs	1863 (9.7)
ACEi/ARB^∗^ + CCB + Diuretics^1^ + MRA	194 (10.4)

*Note:* Unknown and missing data were excluded from percentage calculations. Where indicated *n* represents the number of patients available data.

Abbreviations: 2D, 2‐dimensional; ACEi, angiotensin converting enzyme inhibitors; ARBs, angiotensin receptor blockers; BMI, body mass index; CCBs, calcium channel blockers; CKD‐EPI 2021, Chronic Kidney Disease Epidemiology Collaboration 2021 eGFR equation; CKDs, chronic kidney diseases; CVDs, cardiovascular diseases; DBP, diastolic blood pressure; ECG, electrocardiogram; eGFR, estimated glomerular filtration rate; Hb1Ac, glycated hemoglobin; HF, heart failure; LAE, left atrial enlargement; LDL‐c, low‐density lipoprotein cholesterol; LVEF; left ventricular ejection fraction; LVH, left ventricular hypertrophy; MRA, mineralocorticoid receptor antagonists; SBP, systolic blood pressure; SD, standard deviation; T2DM, Type 2 diabetes mellitus; uACR, urine albumin–creatinine ratio.

^#^All data presented as *n* (%) unless specified.

^∗^Proportion of patients receiving angiotensin receptor/neprilysin inhibitors were included among the ARB category.

^1^Include thiazide diuretics, thiazide‐like diuretics, loop diuretics, potassium sparing diuretics except MRA.

The mean (SD) age was 60.3 (12.04) years, with the mean (SD) HTN duration of 9.8 (8.63) years. Just over half (51.9%) were female, nearly 7 in 10 (69.3%) had at least secondary education, approximately 2 in 5 (42.5%) were employed, and more than three‐quarters (77.0%) reported health insurance coverage.

Prevalent comorbidities included T2DM in 16,458 (71.4%) patients (mean HbA1c: 7.9%), CKD in 8803 (38.2%) patients (mean eGFR: 43.3 mL/min/1.73 m^2^), HF in 3371 (14.6%) patients (mean left ventricular ejection fraction [LVEF]: 42.1%), while 2566 (11.1%) patients had none of these comorbidities.

### 3.1. Overall Population: Clinical Evaluation, Diagnostic Procedures, and Laboratory Test

#### 3.1.1. Cardiovascular Risk Factors

The cohort carried a substantial burden of cardiovascular risk factors; 38.0% were aged ≥ 65 years, 34.3% with a family history of CVD, and 20.1% were smokers or had history of smoking. Obesity (BMI ≥ 30 kg/m^2^) affected just over two‐fifths of participants (40.5%), and more than half had a history of dyslipidemia (51.0%). Additionally, the mean (SD) HbA1c was 7.5 (1.97) % and the mean (SD) low‐density lipoproteins cholesterol (LDL‐c) was 96.6 (43.96) mg/dL.

#### 3.1.2. Hypertension‐Mediated Organ Damage (HMOD)

2D ECHO results were available for 6602 (28.6%) subjects, of whom 30.6% exhibited left ventricular hypertrophy (LVH) and 42.4% diastolic dysfunction.

Serum creatinine and uACR were measured in 16,474 (71.4%) and 8694 (37.7%) individuals, respectively, reporting CKD stage G3 (eGFR 30–59 mL/min/1.73 m^2^) in 24.0% and albuminuria stage A2 (uACR 30–300 mg/g) in 32.1%.

#### 3.1.3. Established Cardiovascular and Kidney Disease

Among participants with available records, 18.0% had a history of coronary artery disease, while 4.4% had experienced a prior stroke. Among those with kidney function assessment, 17.9% had advanced CKD (stage G4 and G5, eGFR < 30 mL/min/1.73 m^2^), and 18.1% had albuminuria stage A3 (uACR > 300 mg/g). Atrial fibrillation was present in 6.9% of participants with available ECG results.

### 3.2. Overall Population: Pharmacological Management of HTN

Antihypertensive medications were prescribed to 88.7% of patients. RAS blockers were the most frequently prescribed drug class, accounting for more than three‐quarters of prescriptions (76.6%), followed by CCBs (42.0%), beta‐blockers (41.4%), and diuretics (23.7%).

The majority of patients (62.3%) were prescribed combination therapy (≥ 2 antihypertensive agents). Of these, 33.0% received dual therapy, most commonly a RAS blocker combined with a CCB or a diuretic (50.4% of dual‐therapy prescriptions), while 19.6% were prescribed triple therapy, with the RAS blocker/CCB/diuretic combination representing the predominant regimen (17.6% of triple‐therapy prescriptions).

### 3.3. Overall Population: BP Levels and Burden of Uncontrolled HTN

Office BP measurements were documented for 94.7% of participants, with a mean (SD) SBP of 134.6 (19.91) mmHg and a mean (SD) DBP of 79.7 (12.46) mmHg. Applying the more stringent target for control (SBP < 130 mmHg and DBP < 80 mmHg), which aligns with most recent guidelines, more than three‐quarters (75.4%) of the cohort had uncontrolled HTN. When using the historical BP target of SBP < 140 mmHg and DBP < 90 mmHg, uncontrolled HTN was still observed in 46.0% of participants.

### 3.4. Patient Characteristics and Treatment Patterns Grouped by Comorbidity

Table 2 details the demographic, clinical, and pharmacological characteristics of the study cohort, stratified into mutually exclusive comorbidity groups: T2DM, CKD, HF, and the absence of these comorbidities (HTN alone group).

**TABLE 2:** Demographic, clinical characteristics, and treatment patterns of the study populations grouped by mutually exclusive comorbidities.

Characteristics^#^	HTN + T2DM alone [*N* = 9988]	HTN + CKD alone [*N* = 2664]	HTN + HF alone [*N* = 690]	HTN alone [*N* = 2566]	*p*‐value^$^
Age, *n*	9973	2661	689	2566	
Mean (SD), years	59.2 (10.51)	56.8 (16.11)	61.1 (12.98)	59.2 (12.67)	< 0.0001
Female	5645 (56.5)	1240 (46.5)	251 (36.4)	1543 (60.1)	< 0.0001
Duration of HTN, *n*	6892	2317	546	1892	
Mean (SD), years	9.2 (8.12)	9.4 (9.13)	8.7 (8.30)	9.8 (9.22)	0.0239
Comorbidities					
Duration of T2DM	8342	NA	NA	NA	
Mean (SD), years	9.8 (8.12)	NA	NA	NA	
Duration of CKD	NA	1885	NA	NA	
Mean (SD), years	NA	5.2 (6.11)	NA	NA	
Duration of HF	NA	NA	499	NA	
Mean (SD), years	NA	NA	2.9 (3.33)	NA	
Cardiovascular Risk Factors					
Age (≥ 65 years)	3099 (31.1)	942 (35.4)	294 (42.7)	931 (36.3)	< 0.0001
Family History of CVD	2828 (36.6)	420 (19.8)	238 (40.8)	797 (46.8)	< 0.0001
Current smoker	664 (7.1)	169 (6.7)	86 (12.7)	144 (6.0)	< 0.0001
Ex‐smoker	993 (10.6)	295 (11.7)	141 (20.8)	255 (10.7)	< 0.0001
Obesity (BMI ≥ 30 kg/m^2^)	4045 (45.1)	567 (27.1)	195 (34.3)	922 (43.5)	< 0.0001
History of Dyslipidemia	4515 (55.1)	749 (33.7)	208 (36.3)	924 (43.8)	< 0.0001
HbA1c, *n*	8477	1156	236	957	
Mean (SD), %	8.0 (1.96)	5.6 (0.89)	6.0 (1.14)	6.1 (1.42)	< 0.0001
LDL‐c, *n*	7146	1538	313	1190	
Mean (SD), mg/dL	94.4 (43.46)	107.5 (46.11)	98.6 (46.35)	107.4 (40.75)	< 0.0001
Hypertension‐Mediated Organ damage					
2D Echocardiography, *n*	1867 (18.7)	478 (17.9)	631 (91.4)	850 (33.1)	
LVH	328 (17.6)	208 (43.5)	211 (33.4)	266 (31.3)	< 0.0001
Diastolic dysfunction	438 (23.5)	206 (43.1)	400 (63.4)	345 (40.6)	< 0.0001
LAE	163 (8.7)	73 (15.3)	336 (53.2)	157 (18.5)	< 0.0001
eGFR (CKD‐EPI 2021) , *n*	5382	2498	546	1667	
eGFR 30–59 mL/min/1.73 m^2^	288 (5.4)	974 (39.0)	31 (5.7)	33 (2.0)	< 0.0001
uACR, *n*	4561	801	33	126	
uACR 30–300 mg/g	1002 (22.0)	292 (36.5)	10 (30.3)	3 (2.4)	< 0.0001
Established CVD and CKD					
History of coronary artery disease	603 (9.3)	202 (8.9)	272 (44.8)	278 (13.9)	< 0.0001
History of stroke	191 (2.9)	97 (4.0)	45 (7.1)	78 (3.7)	< 0.0001
eGFR < 30 mL/min/1.73 m^2^	67 (1.2)	1199 (48.0)	7 (1.3)	16 (1.0)	< 0.0001
uACR > 300 mg/g	249 (5.5)	308 (38.5)	7 (21.2)	1 (0.8)	< 0.0001
ECG, *n*	2171 (66.7)	565 (31.8)	562 (87.0)	1275 (69.0)	
Atrial fibrillation	39 (1.8)	20 (3.5)	92 (16.4)	38 (3.0)	< 0.0001
Office Blood pressure Measurements (n)	9423	2443	668	2503	
Mean (SD) SBP, mmHg	132.3 (17.60)	140.8 (22.41)	126.4 (21.63)	134.6 (18.57)	< 0.0001
Mean (SD) DBP, mmHg	79.1 (11.18)	84.4 (14.93)	78.4 (13.34)	81.2 (12.36)	< 0.0001
SBP ≥ 130 mmHg or DBP ≥ 80 mmHg	7047 (74.8)	2049 (83.9)	398 (59.6)	1902 (76.0)	< 0.0001
SBP ≥ 140 mmHg or DBP ≥ 90 mmHg	3857 (40.9)	1468 (60.1)	237 (35.5)	1176 (47.0)	< 0.0001
Treatment Patterns, *n*	9209	2550	667	2380	
Antihypertensive medications	7550 (82.0)	2492 (97.7)	664 (99.6)	2273 (95.5)	
ACEi/ARB^∗^	6276 (83.1)	1427 (57.3)	559 (84.2)	1794 (78.9)	< 0.0001
Alpha blockers	71 (0.9)	158 (6.3)	5 (0.8)	18 (0.8)	< 0.0001
Beta blockers	2088 (27.7)	1148 (46.1)	567 (85.4)	778 (34.2)	< 0.0001
CCB	2655 (35.2)	1477 (59.3)	81 (12.2)	1070 (47.1)	< 0.0001
Central acting agents	103 (1.4)	236 (9.5)	1 (0.2)	86 (3.8)	< 0.0001
Diuretics^1^	925 (12.3)	577 (23.2)	349 (52.6)	514 (22.6)	< 0.0001
Vasodilators	66 (0.9)	157 (6.3)	12 (1.8)	39 (1.7)	< 0.0001
MRA	188 (2.5)	91 (3.7)	396 (59.6)	144 (6.3)	< 0.0001
Renin‐Inhibitors	2 (0.0)	0	0	0	
Antihypertensive regimens					
1 antihypertensive drug	4035 (53.4)	779 (31.3)	48 (7.2)	843 (37.1)	< 0.0001
2 antihypertensive drugs	2424 (32.1)	946 (38.0)	153 (23.0)	843 (37.1)	< 0.0001
ACEi/ARB^∗^ + CCB	1202 (49.6)	270 (28.5)	7 (4.6)	342 (40.6)	< 0.0001
ACEi/ARB^∗^ + Diuretics^1^	296 (12.2)	102 (10.8)	7 (4.6)	121 (14.4)	0.0032
3 antihypertensive drugs	849 (11.2)	519 (20.8)	251 (37.8)	440 (19.4)	< 0.0001
ACEi/ARB^∗^ + CCB + Diuretics^1^	191 (22.5)	96 (18.5)	2 (0.8)	124 (28.2)	< 0.0001
≥ 4 antihypertensive drugs	242 (3.2)	248 (10.0)	212 (31.9)	147 (6.5)	< 0.0001
ACEi/ARB^∗^+CCB + Diuretics^1^ + MRA	34 (14.0)	12 (4.8)	16 (7.5)	28 (19.0)	< 0.0001

*Note:* Unknown and missing data were excluded from percentage calculations. Where indicated *n* represents the number of patients available data.

Abbreviations: 2D, 2‐dimensional; ACEi, angiotensin converting enzyme inhibitors; ARBs, angiotensin receptor blockers; BMI, body mass index; CCBs, calcium channel blockers; CKD‐EPI 2021, Chronic Kidney Disease Epidemiology Collaboration eGFR equation; CKDs, chronic kidney diseases; CVDs, cardiovascular diseases; DBP, diastolic blood pressure; ECG, electrocardiogram; eGFR, estimated glomerular filtration rate; Hb1Ac, glycated hemoglobin; HF, heart failure; LAE, left atrial enlargement; LDL‐c, low‐density lipoprotein cholesterol; LVH, left ventricular hypertrophy; MRA, mineralocorticoid receptor antagonists; SBP, systolic blood pressure; SD, standard deviation; T2DM, Type 2 diabetes mellitus; uACR, urine albumin–creatinine ratio.

^#^All data presented as *n* (%) unless specified.

^∗^Proportion of patients receiving angiotensin receptor/neprilysin inhibitors were included among the ARB category.

^1^Include thiazide diuretics, thiazide‐like diuretics, loop diuretics, potassium sparing diuretics except MRA.

^$^ *p*‐value was calculated using ANOVA for continuous variables and Chi‐square/Fisher’s exact test for categorical variables.

Across comorbidity subgroups, risk profiles and BP control varied markedly. Participants with HTN + T2DM had the highest prevalence of dyslipidemia (55.1% of participants) and obesity (BMI ≥ 30 kg/m^2^; 45.1% of participants) compared with HTN + CKD, HTN + HF, and HTN alone. Those with HTN + CKD carried the greatest burden of LVH (43.5% of participants) and the highest proportion of uncontrolled HTN (83.9% of participants at SBP ≥ 130 mmHg or DBP ≥ 80 mmHg), followed by HTN alone (76.0%), HTN + T2DM (74.8%), and HTN + HF (59.6%). The HTN + HF subgroup was older (mean age 61.1 years) and more frequently male (63.6% of participants) than HTN + CKD (56.8 years; 53.5% male), HTN + T2DM (59.2 years; 43.5% male), or HTN alone (59.2 years; 39.9% male). Furthermore, the HTN + HF subgroup had the highest rates of diastolic dysfunction (63.4% of participants), left atrial enlargement (53.2%), atrial fibrillation (16.4%), prior coronary artery disease (44.8%), and prior stroke (7.1%). In contrast, participants with HTN alone had the highest prevalence of family history of CVD (46.8% of participants).

Distinct patterns in pharmacological management emerged across the groups. The proportion of patients receiving antihypertensive medication was highest in the HTN + HF group (99.6%), followed by HTN + CKD (97.7%) and HTN alone (95.5%), and was lowest in the HTN + T2DM group (82.0%). Patients with HTN + HF had greater use of RAS blockers (84.2%), beta‐blockers (85.4%), diuretics (52.6%), and MRAs (59.6%), whereas those with HTN + CKD had the highest rate of CCB prescription (59.3%). Regarding treatment intensity, a higher proportion of patients in the HTN + HF group received combination therapy (92.8%), while monotherapy was most common in the HTN + T2DM group (53.4%).

### 3.5. Patient Characteristics and Treatment Patterns Grouped by Region

Table 3 details the demographic, clinical, and pharmacological characteristics of the study cohort, stratified by geographic region.

**TABLE 3:** Demographic, clinical characteristics, and treatment patterns of the study populations grouped by geographic regions.

Characteristics^#^	MEA [*N* = 9794]	APAC [*N* = 6292]	LATAM [*N* = 6170]	Eurasia [*N* = 807]	*p*‐value^$^
Age, *n*	9773	6282	6167	805	
Mean (SD), years	59.0 (12.52)	59.9 (11.12)	62.1 (11.96)	63.8 (11.35)	< 0.0001
Female	5143 (52.5)	2978 (47.3)	3476 (56.3)	369 (45.7)	< 0.0001
Duration of HTN, *n*	7532	3712	5089	600	
Mean (SD), years	9.5 (7.85)	9.1 (8.03)	11.2 (9.83)	6.7 (8.67)	< 0.0001
Comorbidities					
History of T2DM	5959 (60.8)	5810 (92.3)	4149 (67.2)	540 (66.9)	< 0.0001
History of CKD	4467 (45.6)	2181 (34.7)	1841 (29.8)	314 (38.9)	< 0.0001
History of HF	1986 (20.3)	387 (6.2)	694 (11.2)	304 (37.7)	< 0.0001
Cardiovascular Risk Factors					
Age (≥ 65 years)	3433 (35.1)	2227 (35.5)	2681 (43.5)	414 (51.4)	< 0.0001
Family History of CVD	1996 (25.8)	1495 (34.8)	2088 (44.7)	308 (64.7)	< 0.0001
Current smoker	903 (9.6)	282 (5.0)	422 (7.4)	52 (6.8)	< 0.0001
Ex‐smoker	955 (10.2)	494 (8.7)	1155 (20.2)	60 (7.8)	< 0.0001
Obesity (BMI ≥ 30 kg/m^2^)	3577 (46.4)	1523 (27.5)	2371 (43.2)	439 (57.0)	< 0.0001
History of Dyslipidemia	3223 (40.1)	3103 (65.1)	2697 (50.9)	573 (78.7)	< 0.0001
HbA1c, *n*	6477	5419	3906	675	< 0.0001
Mean (SD), %	7.4 (1.98)	7.8 (1.83)	7.5 (2.10)	7.3 (1.92)	
LDL‐c, *n*	6022	4522	3989	620	< 0.0001
Mean (SD), mg/dL	103.3 (44.80)	88.2 (41.06)	93.2 (43.20)	114.7 (45.70)	
Hypertension‐Mediated Organ damage					
2D Echocardiography, *n*	3484	527	2112	479	
LVH	1026 (29.4)	162 (30.7)	468 (22.2)	364 (76.0)	< 0.0001
Diastolic dysfunction	1667 (47.8)	183 (34.7)	656 (31.1)	290 (60.5)	< 0.0001
LAE	1078 (30.9)	122 (23.1)	410 (19.4)	195 (40.7)	< 0.0001
eGFR (CKD‐EPI 2021) , *n*	6939	4443	4347	745	
eGFR 30–59 mL/min/1.73 m^2^	1804 (26.0)	1004 (22.6)	955 (22.0)	192 (25.8)	< 0.0001
uACR, *n*	2947	3019	2324	404	
uACR 30–300 mg/g	959 (32.5)	1143 (37.9)	576 (24.8)	109 (27.0)	< 0.0001
Established CVD and CKD					
History of coronary artery disease	1733 (22.7)	465 (13.9)	538 (10.4)	299 (41.5)	< 0.0001
History of stroke	326 (4.0)	135 (4.1)	222 (4.3)	86 (12.1)	< 0.0001
eGFR < 30 mL/min/1.73 m^2^	1778 (25.6)	486 (10.9)	665 (15.3)	18 (2.4)	< 0.0001
uACR > 300 mg/g	778 (26.4)	529 (17.5)	257 (11.1)	8 (2.0)	< 0.0001
ECG, *n*	3619	1402	1861	593	
Atrial fibrillation	315 (8.7)	36 (2.6)	72 (3.9)	90 (15.2)	< 0.0001
Office Blood pressure Measurements	9503 (97.0)	5823 (92.5)	5719 (92.7)	792 (98.1)	
Mean (SD) SBP, mmHg	137.1 (20.58)	134.7 (19.15)	129.6 (18.61)	140.3 (18.80)	< 0.0001
Mean (SD) DBP, mmHg	81.7 (12.96)	78.4 (12.07)	77.1 (11.38)	83.9 (11.31)	< 0.0001
SBP ≥ 130 mmHg or DBP ≥ 80 mmHg	7608 (80.1)	4258 (73.1)	3925 (68.6)	671 (84.7)	< 0.0001
SBP ≥ 140 mmHg or DBP ≥ 90 mmHg	5062 (53.3)	2553 (43.8)	1976 (34.6)	446 (56.3)	< 0.0001
Treatment Patterns, *n*	9522	5905	5432	795	
Antihypertensive medications	8678 (91.1)	4773 (80.8)	5028 (92.6)	726 (91.3)	< 0.0001
ACEi/ARB^∗^	5883 (67.8)	3749 (78.5)	4505 (89.6)	568 (78.2)	< 0.0001
Alpha blockers	353 (4.1)	186 (3.9)	40 (0.8)	4 (0.6)	< 0.0001
Beta blockers	4377 (50.4)	1595 (33.4)	1504 (29.9)	473 (65.2)	< 0.0001
CCB	3982 (45.9)	2193 (45.9)	1648 (32.8)	236 (32.5)	< 0.0001
Central acting agents	439 (5.1)	77 (1.6)	97 (1.9)	9 (1.2)	< 0.0001
Diuretics^1^	2634 (30.4)	738 (15.5)	954 (19.0)	235 (32.4)	< 0.0001
Vasodilators	373 (4.3)	36 (0.8)	43 (0.9)		< 0.0001
MRA	1043 (12.0)	250 (5.2)	449 (8.9)	169 (23.3)	< 0.0001
Renin‐Inhibitors	2 (0.0)		1 (0.0)		
Antihypertensive regimens					
1 antihypertensive drug	2666 (30.7)	2060 (43.2)	2317 (46.1)	190 (26.2)	< 0.0001
2 antihypertensive drugs	2844 (32.8)	1713 (35.9)	1558 (31.0)	228 (31.4)	< 0.0001
ACEi/ARB^∗^ + CCB	889 (31.3)	840 (49.0)	661 (42.4)	43 (18.9)	< 0.0001
ACEi/ARB^∗^ + Diuretics^1^	303 (10.7)	177 (10.3)	249 (16.0)	29 (12.7)	< 0.0001
3 antihypertensive drugs	2050 (23.6)	710 (14.9)	804 (16.0)	202 (27.8)	< 0.0001
ACEi/ARB^∗^ + CCB + Diuretics^1^	302 (14.7)	144 (20.3)	182 (22.6)	34 (16.8)	< 0.0001
≥ 4 antihypertensive drugs	1118 (12.9)	290 (6.1)	349 (6.9)	106 (14.6)	< 0.0001
ACEi/ARB^∗^ + CCB + Diuretics^1^ + MRA	89 (8.0)	42 (14.5)	44 (12.6)	19 (17.9)	0.0001

*Note:* Unknown and missing data were excluded from percentage calculations. Where indicated *n* represents the number of patients available data.

Abbreviations: 2D, 2‐dimensional; ACEi, angiotensin converting enzyme inhibitors; ARBs, angiotensin receptor blockers; BMI, body mass index; CCBs, calcium channel blockers; CKD‐EPI, Chronic Kidney Disease Epidemiology Collaboration eGFR equation; CKDs, chronic kidney diseases; CVDs, cardiovascular diseases; DBP, diastolic blood pressure; ECG, electrocardiogram; eGFR, estimated glomerular filtration rate; Hb1Ac, glycated hemoglobin; HF, heart failure; LAE, left atrial enlargement; LDL‐c, low‐density lipoprotein cholesterol; LVH, left ventricular hypertrophy; MRA, mineralocorticoid receptor antagonists; SBP, systolic blood pressure; SD, standard deviation; T2DM, Type 2 diabetes mellitus; uACR, urine albumin–creatinine ratio.

^#^All data presented as *n* (%) unless specified.

^∗^Proportion of patients receiving angiotensin receptor/neprilysin inhibitors were included among the ARB category.

^1^Include thiazide diuretics, thiazide‐like diuretics, loop diuretics, potassium sparing diuretics except MRA.

^$^ *p*‐value was calculated using ANOVA for continuous variables and Chi‐square/Fisher’s exact test for categorical variables.

Participants from Eurasia were older (mean age, 63.8 years) and included a greater share of males (54.3%) compared to those from APAC, LATAM, or MEA. Moreover, the Eurasia group exhibited the highest prevalence of dyslipidemia (78.7%), obesity (57.0%), family history of CVD (64.7%), coronary artery disease (41.5%), prior stroke (12.1%), comorbid HF (37.7%), and atrial fibrillation (15.2%). Furthermore, echocardiographic abnormalities—including LVH (76.0%), diastolic dysfunction (60.5%), and left atrial enlargement (40.7%)—were most frequent in this region. BP control was poorest in Eurasia, with 84.7% of participants having SBP ≥ 130 mmHg or DBP ≥ 80 mmHg. In contrast, participants from the MEA region reported the highest proportion of comorbid CKD (45.6%), while those from APAC registered the highest prevalence of T2DM (92.3%).

Antihypertensive medication use was broadly comparable across regions (80.8%–92.6%). ACEi/ARB was the most prescribed class in all regions, with the highest utilization observed in LATAM (89.6%) and the lowest in MEA (67.8%). The majority of patients in Eurasia (73.8%) and MEA (69.3%) received combination therapy (≥ 2 agents), whereas monotherapy predominated in APAC (43.2%) and LATAM (46.1%).

### 3.6. Patient Characteristics and Treatment Patterns Grouped by HTN Control Status

Table 4 details the demographic, clinical, and therapeutic characteristics of the population with BP measurements stratified by HTN control status.

**TABLE 4:** Demographic, clinical characteristics, and treatment patterns of the patients with BP data grouped by level of control.

Characteristics^#^	Controlled HTN group [*N* = 5375]	Uncontrolled HTN group [*N* = 16,462]	Total with BP [*N* = 21,837]	*p*‐value^$^
Age, *n*	5362	1,6440	21,802	
Mean (SD), years	61.8 (11.57)	59.8 (12.09)	60.3 (11.99)	< 0.0001
Female	2734 (50.9)	8615 (52.3)	11,349 (52.0)	0.0615
Duration of HTN, *n*	3783	12,582	16,365	
Mean (SD), years	10.1 (8.78)	9.7 (8.57)	9.8 (8.62)	0.0038
Comorbidities				
History of T2DM	3938 (73.3)	11,622 (70.6)	15,560 (71.3)	0.0002
History of CKD	1780 (33.1)	6467 (39.3)	8247 (37.8)	< 0.0001
History of HF	1056 (19.6)	2222 (13.5)	3278 (15.0)	< 0.0001
Cardiovascular Risk Factors				
Age (≥ 65)	2265 (42.2)	6060 (36.9)	8325 (38.2)	< 0.0001
Family History of CVD	1407 (36.4)	4132 (32.9)	5539 (33.7)	< 0.0001
Current smoker	443 (8.7)	1132 (7.4)	1575 (7.7)	< 0.0018
Ex‐smoker	742 (14.6)	1825 (11.9)	2567 (12.6)	< 0.0001
Obesity (BMI ≥ 30 kg/m^2^)	1630 (34.6)	5989 (42.5)	7619 (40.5)	< 0.0001
History of Dyslipidemia	2403 (57.0)	6837 (49.6)	9240 (51.3)	< 0.0001
HbA1c	3736	12,081	1,5817	0.2608
Mean (SD)	7.5 (1.92)	7.6 (1.98)	7.5 (1.97)	
LDL‐c (mg/dL)	3495	11,084	14,579	< 0.0001
Mean (SD)	88.1 (38.68)	99.3 (44.93)	96.6 (43.77)	
Hypertension‐Mediated Organ damage				
2D Echocardiography	1893	4580	6473	
LVH	470 (24.8)	1506 (32.9)	1976 (30.5)	< 0.0001
Diastolic dysfunction	802 (42.4)	1945 (42.5)	2747 (42.4)	0.0343
LAE	608 (32.1)	1180 (25.8)	1788 (27.6)	< 0.0001
eGFR (CKD‐EPI 2021)	4031 (75.0)	11,857 (72.0)	15,888 (72.8)	
eGFR 30–59 mL/min/1.73 m^2^	1029 (25.5)	2815 (23.7)	3844 (24.4)	0.0222
uACR	1905 (35.4)	6564 (39.9)	8469 (38.8)	
uACR 30–300 mg/g	591 (31.0)	2126 (32.4)	2717 (32.1)	0.2611
Established CVD and CKD				
History of coronary artery disease	890 (23.6)	2077 (16.9)	2967 (18.5)	< 0.0001
History of stroke	175 (4.5)	568 (4.5)	743 (4.5)	0.8673
eGFR < 30 mL/min/1.73 m^2^	435 (10.8)	2282 (19.2)	2717 (17.1)	< 0.0001
uACR > 300 mg/g	272 (14.3)	1268 (19.3)	1540 (18.2)	< 0.0001
ECG, *n*	2218	5111	7329	
Atrial fibrillation	184 (8.3)	323 (6.3)	507 (6.9)	0.2103
Office Blood pressure Measurements				
Mean (SD) SBP, mmHg	114.0 (9.76)	141.3 (17.63)	134.6 (19.91)	< 0.0001
Mean (SD) DBP, mmHg	67.4 (6.84)	83.7 (11.18)	79.7 (12.46)	< 0.0001
Treatment Patterns, *n*	4972	11,235	16,207	
Antihypertensive medications	4411 (88.7)	6768 (60.2)	11,179 (69.0)	< 0.0001
ACEi/ARB^∗^	3516 (79.7)	4672 (69.0)	8188 (73.2)	< 0.0001
Alpha blockers	86 (1.9)	139 (2.1)	225 (2.0)	0.7016
Beta blockers	1936 (43.9)	2370 (35.0)	4306 (38.5)	< 0.0001
CCB	1458 (33.1)	2613 (38.6)	4071 (36.4)	< 0.0001
Central acting agents	68 (1.5)	105 (1.6)	173 (1.5)	0.9672
Diuretics^1^	1164 (26.4)	1149 (17.0)	2313 (20.7)	< 0.0001
Vasodilators	51 (1.2)	79 (1.2)	130 (1.2)	0.9575
MRA	729 (16.5)	458 (6.8)	1187 (10.6)	< 0.0001
Renin‐Inhibitors	2 (0.0)		2 (0.0)	
Antihypertensive regimens				
1 antihypertensive drug	1686 (38.2)	3639 (53.8)	5325 (47.6)	< 0.0001
2 antihypertensive drugs	1371 (31.1)	1855 (27.4)	3226 (28.9)	< 0.0001
ACEi/ARB^∗^ + CCB	500 (36.5)	773 (41.7)	1273 (39.5)	0.0028
ACEi/ARB^∗^ + Diuretics^1^	184 (13.4)	234 (12.6)	418 (13.0)	0.5002
3 antihypertensive drugs	857 (19.4)	881 (13.0)	1738 (15.5)	< 0.0001
ACEi/ARB^∗^ + CCB + Diuretics^1^	112 (13.0)	194 (22.0)	306 (17.6)	< 0.0001
≥ 4 antihypertensive drugs	497 (11.3)	393 (5.8)	890 (8.0)	< 0.0001
ACEi/ARB^∗^ + CCB + Diuretics^1^ + MRA	53 (10.7)	43 (10.9)	96 (10.8)	0.8946

*Note:* Unknown and missing data were excluded from percentage calculations. Where indicated *n* represents the number of patients available data.

Abbreviations: 2D, 2‐dimensional; ACEi, angiotensin converting enzyme inhibitors; ARBs, angiotensin receptor blockers; BMI, body mass index; CCBs, calcium channel blockers; CKD‐EPI 2021, Chronic Kidney Disease Epidemiology Collaboration eGFR equation; CKDs, chronic kidney diseases; CVDs, cardiovascular diseases; DBP, diastolic blood pressure; ECG, electrocardiogram; eGFR, estimated glomerular filtration rate; Hb1Ac, glycated hemoglobin; HF, heart failure; LAE, left atrial enlargement; LDL‐c, low‐density lipoprotein cholesterol; LVH, left ventricular hypertrophy; MRA, mineralocorticoid receptor antagonists; SBP, systolic blood pressure; SD, standard deviation; T2DM, Type 2 diabetes mellitus; uACR, urine albumin–creatinine ratio.

^#^All data presented as *n* (%) unless specified.

^∗^Proportion of patients receiving angiotensin receptor/neprilysin inhibitors were included among the ARB category.

^1^Include thiazide diuretics, thiazide‐like diuretics, loop diuretics, potassium sparing diuretics except MRA.

^$^ *p*‐value calculated considering controlled HTN and uncontrolled HTN groups using *t*‐test for continuous variables and Chi‐square/Fisher’s exact test for categorical variables.

A total of 21,837 participants were analyzed, comprising 5375 with controlled HTN (SBP < 130 mmHg and DBP < 80 mmHg) and 16,462 with uncontrolled HTN (SBP ≥ 130 mmHg or DBP ≥ 80 mmHg). Within the uncontrolled HTN cohort, the mean (SD) age was 59.8 (12.09) years, with 52.3% being female. The mean (SD) duration of HTN was 9.7 (8.57) years, and the mean (SD) SBP/DBP was 141.3 (17.63)/83.7 (11.18) mmHg.

The examination of cardiovascular risk factors within the uncontrolled HTN cohort revealed the presence of various predisposing conditions. Over one‐third (36.9%) were aged ≥ 65 years; 32.9% reported a family history of CVD, and current or former smoking habits were noted in 19.3% of this group. Obesity prevailed in 42.5% of the cohort, nearly half (49.6%) had a history of dyslipidemia, and 70.6% had comorbid T2DM. Additionally, the mean (SD) HbA1c for this group was 7.6 (1.98) %, and the mean (SD) LDL‐c was 99.3 (44.93) mg/dL.

Assessment of HMOD indicated notable findings within the uncontrolled HTN cohort. Among those with available 2D ECHO results (*n* = 4580), LVH was reported in 32.9% and diastolic dysfunction in 42.5% of participants. Serum creatinine and uACR were measured in 11,857 (72.0%) and 6564 (39.9%) subjects, respectively, revealing CKD stage G3 (eGFR 30–59 mL/min/1.73 m^2^) in 23.7% and albuminuria stage A2 (uACR 30–300 mg/g) in 32.4%.

For established cardiovascular and kidney diseases, data revealed that 16.9% of participants with available records in the uncontrolled HTN group had a history of coronary artery disease, and 4.5% reported a previous stroke. Advanced CKD, defined as eGFR stages G4 and G5 (eGFR < 30 mL/min/1.73 m^2^), was observed in 19.2%, and albuminuria stage A3 (uACR > 300 mg/g) was identified in 19.3%. Atrial fibrillation was noted in 6.3% of those with ECG results.

Regarding treatment patterns, only 60.2% of patients with uncontrolled HTN were prescribed antihypertensive medication. RAS blockers (69.0%) constituted the most frequently utilized class.

Monotherapy predominated with 53.8% of uncontrolled HTN patients. Dual therapy was administered to 27.4% of the cohort and 13.0% were on triple therapy. Among those on dual therapy, ACEi/ARB combined with CCB or diuretics accounted for 54.3%. Among those receiving triple therapy, the combination of ACEi/ARB, CCB, and diuretic was used in only 22.0% of cases.

Compared to the controlled HTN group, patients in the uncontrolled HTN group were, on average, younger and exhibited a shorter mean duration of HTN. Furthermore, they had a higher prevalence of obesity, a greater rate of LVH, and a higher proportion of comorbid CKD. Additionally, the uncontrolled HTN cohort also presented with increased mean LDL‐c levels compared to the controlled HTN group.

In terms of pharmacological management, a notably lower proportion of patients in the uncontrolled HTN group were prescribed antihypertensive medication and they showed a higher tendency toward monotherapy compared to the controlled HTN group.

### 3.7. Determinants of Uncontrolled HTN (Logistic Regression Analysis)

**FIGURE 1:**
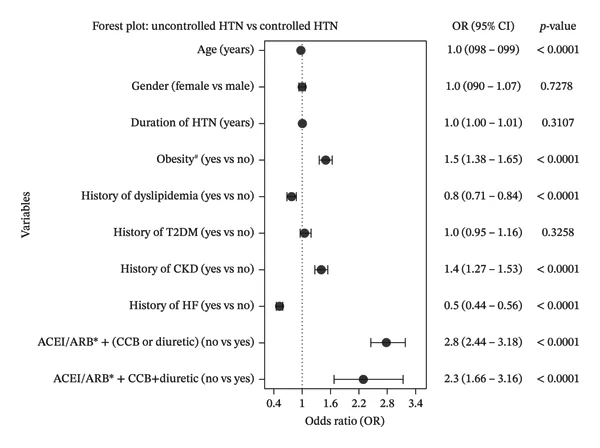
Multivariate analysis of factors associated with uncontrolled HTN. Data were analyzed by multivariate regression analysis. *p*‐value < 0.05 is considered statistically significant. ^#^BMI ≥ 30 kg/m^2^. ^∗^Proportion of patients receiving angiotensin receptor/neprilysin inhibitors were included among the ARB category. ACEi, angiotensin converting enzyme inhibitors; ARBs, angiotensin receptor blockers; BMI, body mass index; CCBs, calcium channel blockers; CKD, chronic kidney disease; HF, heart failure; HTN, hypertension; OR, odds ratio; T2DM, Type 2 diabetes mellitus.

In multivariable logistic regression (Figure 1), nonuse of guideline‐recommended RAS blocker‐based combinations was independently associated with uncontrolled HTN for both dual therapy (ACEi/ARB + CCB or diuretic; OR [95% CI]: 2.8 [2.44–3.18]; *p* < 0.0001) and triple therapy (ACEi/ARB + CCB + diuretic; OR [95% CI]: 2.3 [1.66–3.16]; *p* < 0.0001). Obesity (OR [95% CI]: 1.5 [1.38–1.65]; *p* < 0.0001) and history of CKD (OR [95% CI]: 1.4 [1.27–1.53]; *p* < 0.0001) were also independently associated with uncontrolled HTN.

## 4. Discussion

The iCaReMe Global Registry offers a distinctive perspective on the real‐world burden of uncontrolled HTN through a cross‐sectional analysis of baseline data from 23,063 participants with HTN, drawn from a broader CVRM cohort enrolled between February 2018 and October 2024 across 29 countries. The extensive geographic representation, spanning diverse regions such as APAC, Eurasia, LATAM, and MEA, captures a heterogeneous patient profile reflective of varied genetic, socioeconomic, and healthcare access backgrounds, a feature often underrepresented in HTN research, which tends to focus on high‐income settings [19, 20].

While existing studies often report on HTN prevalence and control in isolated or regionally limited cohorts, comprehensive data on HTN characteristics and management patterns across a global population with CVRM comorbidities are scarce, particularly from LMICs represented in our registry [21]. By encompassing a wide array of geographic and clinical diversity, our analysis sheds light on the complex interplay between HTN and CVRM conditions, where HTN rarely occurs in isolation, amplifying cardiovascular risk through synergistic effects as documented in prior research [9–13, 22].

A central finding from our analysis is the high prevalence of uncontrolled HTN, with more than three‐quarters of participants with available office BP data (75.4% of 21,837 assessed) for the target of SBP < 130 mmHg and DBP < 80 mmHg. Using a higher threshold of SBP < 140 mmHg and DBP < 90 mmHg, nearly half of the cohort (46.0%) still failed to achieve BP goals, indicating the persistent challenge of BP control in routine practice. This mirrors global trends in recent literature, where control rates remain suboptimal, with about 20%–50% of treated hypertensive patients achieving recommended targets worldwide [1, 2, 23, 24].

The substantial burden of comorbid T2DM (71.4%), CKD (38.2%), and HF (14.6%) in the hypertensive cohort illustrates the frequent coexistence of CVRM conditions, complicating HTN management. Stratification by unique comorbidity uncovered distinct patterns, with the HTN + CKD group showing most pronounced prevalence of LVH (43.5%) and uncontrolled HTN (83.9% at SBP/DBP ≥ 130/80 mmHg). This aligns with literature indicating that CKD intensifies HMOD through mechanisms like volume overload and vascular calcification, often leading to resistant HTN [25, 26]. Major global and regional HTN guidelines in their successive editions consistently advocate for early, comprehensive screening for HMOD [14–17, 27–29], yet our data reveal limited diagnostic utilization (e.g., echocardiography available for only 28.6% of the cohort), pointing to implementation barriers across diverse settings.

Geographic stratification exposed disparities in comorbidity burden. Participants from Eurasia exhibited the highest rates of uncontrolled HTN and comorbid HF, while APAC showed the greatest proportion of comorbid T2DM, and MEA had the highest prevalence of comorbid CKD. These regional variations resonate with literature documenting differences in CVRM disease profiles, potentially driven by genetic, environmental, and healthcare access factors [23, 24].

Treatment patterns in our cohort revealed a preference for RAS blockers (76.6% overall), beneficial for patients with T2DM, CKD, and HF due to cardioprotective and renoprotective effects, followed by CCB (42.0%) and beta‐blockers (41.4%) often prioritized in HF management. However, only 60.2% of the uncontrolled HTN group received antihypertensive medication, with a heavy reliance on monotherapy (53.8%) compared to dual (27.4%) or triple therapy (13.0%), contrary to the consensus across 2021 WHO, 2023 ESH, 2024 ESC, and 2025 ACC/AHA guidelines for initial combination pharmacotherapy to enhance control rates [14–17]. Robust evidence supports that combination therapies, especially single‐pill combinations, improve outcomes in patients with CVRM comorbidities [30]. The underuse of combination regimens in our uncontrolled HTN cohort suggests a missed opportunity to address heightened risk profiles, particularly in those with CKD and T2DM, where achieving even the less stringent 140/90‐mmHg target remains challenging for nearly half the cohort.

Analysis by HTN control status showed that the uncontrolled HTN group was younger, had a shorter disease duration, and exhibited higher rates of obesity, LVH, comorbid CKD, and elevated LDL‐c levels compared to the controlled HTN group, alongside reduced antihypertensive medication use and greater reliance on monotherapy. This profile aligns with studies indicating that younger patients and those with comorbidities face adherence or treatment intensification challenges [31]. The increased HMOD in this group emphasizes the urgency of early intervention to prevent progression of CKD and HF, as highlighted by the risk‐based strategies in multiple guidelines.

The multivariable logistic regression revealed the nonuse of RAS blocker–based dual or triple antihypertensive combinations as independent predictors of uncontrolled HTN. These findings align with the 2021 WHO, current 2023 ESH, and 2024 ESC guidelines, which recommend initiating with a RAS blocker combined with a CCB and/or a thiazide diuretic in most hypertensive patients [14–16]. This is further corroborated by a large body of evidence demonstrating that combination regimens achieve superior BP control over monotherapy due to their complementary mechanisms targeting the multifactorial pathophysiology of HTN [30, 32–34].

Our study also identified obesity and CKD as independent comorbid determinants of uncontrolled HTN, consistent with recent observational data [35–37]. Both conditions are recognized to inherently challenge BP control through distinct yet overlapping mechanisms. In obese patients, visceral adiposity drives sympathetic overactivation, hyperleptinemia, and RAAS upregulation, all of which raise the BP setpoint and attenuate antihypertensive response [38]. Similarly, CKD perpetuates HTN through impaired natriuresis, volume overload, and RAAS activity, making BP particularly difficult to control and often requiring intensified multidrug regimens [39].

### 4.1. Strengths and Limitations

Our study offers several notable strengths that enhance its contribution to the field of HTN research within the CVRM context. The iCaReMe Global Registry includes a large and diverse cohort of 23,063 participants across 29 countries, providing a broad representation of hypertensive patients with T2DM, CKD, or HF comorbidities from various healthcare settings. A key strength lies in the originality of our data, which offers contemporary critical insights into HTN clinical characteristics and management patterns in countries and regions (APAC, Eurasia, LATAM, MEA) where such comprehensive data are often limited or unavailable. This addresses a significant gap in literature, as much of the existing research on HTN and CVRM diseases is derived from high‐income settings, often overlooking the unique challenges faced in LMICs represented in our cohort [20]. By capturing real‐world diversity, our analysis provides a foundation for understanding regional disparities and tailoring interventions to underrepresented populations.

Despite these strengths, several limitations must be acknowledged. The cross‐sectional design precludes causal inferences about HTN control and the progression of comorbidities like T2DM, CKD, and HF. Missing data, such as 2D ECHO results for only 28.6% of participants, may underrepresent the true burden of HMOD. Variability in healthcare systems and data collection across regions introduces potential heterogeneity, though this also reflects real‐world diversity. Additionally, as our analysis relies on data records, we could not assess medication adherence, a critical factor influencing BP control and treatment outcomes, which limits our ability to fully interpret the observed underuse of antihypertensive medications and the predominance of monotherapy in the uncontrolled HTN group. Future studies incorporating adherence metrics could provide deeper insights into these treatment gaps.

## 5. Conclusion

In summary, the iCaReMe Global Registry reveals a significant global challenge in HTN control, exacerbated by the high burden of comorbid T2DM, CKD, and/or HF, alongside regional disparities and suboptimal treatment approaches. Our original data from diverse, often understudied regions underscore critical gaps between guideline recommendations and clinical practice across geographies, especially in achieving control targets of < 130/80 mmHg or even < 140/90 mmHg for hypertensive patients with CVRM conditions through early combination therapy and comprehensive HMOD screening. Addressing these gaps with tailored interventions could mitigate the substantial cardiovascular and renal risks observed in this cohort.

## Funding

The study was sponsored by AstraZeneca.

## Conflicts of Interest

Kamlesh Khunti has acted as a consultant, speaker, or received grants for investigator‐initiated studies for Astra Zeneca, Bayer, Novo Nordisk, Sanofi‐Aventis, Servier, Lilly and Merck Sharp & Dohme, Boehringer Ingelheim, Oramed Pharmaceuticals, Pfizer, Roche, Daiichi‐Sankyo, Applied Therapeutics, Embecta, and Nestle Health Science.

Mustafa Arici has received payment or honoraria for lectures, presentations, speaker’s bureaus, advisory boards, steering committee, or educational events from Amgen, Astellas, AstraZeneca, Bayer, Boehringer Ingelheim, CSL Behring, Hilton Pharma, Menarini, Novo.

Carol Pollock has received honoraria for presentations and advisory board roles from Astra Zeneca, Bayer, Eli Lilly, Boehringer Ingelheim, and Otsuka.

Chien‐Ning Huang has received funding for clinical trial from AstraZeneca, Eli Lilly, Novo Nordisk, Sanofi; acted as a consultant, speaker for Abbott, AstraZeneca, Bayer, Boehringer Ingelheim, Eli Lilly, Medtronic, Novartis, Novo Nordisk, Sanofi.

Rubén Silva‐Tinoco reports honoraria for lectures at educational events supported by AstraZeneca, Boehringer Ingelheim, Eli Lilly, Novo Nordisk, and Merck.

Esteban Coto, Karim Salama, Hardik Vasnawala, and Ahmed Hadaoui are AstraZeneca employees.

## Data Availability

Data underlying the findings described in this manuscript may be obtained in accordance with AstraZeneca’s data sharing policy described at https://astrazenecagrouptrials.pharmacm.com/ST/Submission/Disclosure. Data for studies directly listed on Vivli can be requested through Vivli at https://www.vivli.org. Data for studies not listed on Vivli could be requested through Vivli at https://vivli.org/members/enquiries-about-studies-not-listed-on-the-vivli-platform/. AstraZeneca Vivli member page is also available outlining further details: https://vivli.org/ourmember/astrazeneca/

## References

[1] World Health Organization , Global Report on Hypertension 2025: High Stakes—Turning Evidence Into Action, 2025, World Health Organization.

[2] World Health Organization , Hypertension: Fact Sheet, 2025, https://www.who.int/news-room/fact-sheets/detail/hypertension.

[3] Vaduganathan M. et al., The Global Burden of Cardiovascular Diseases and Risk: a Compass for Future Health, Journal of the American College of Cardiology. (2022) 80, no. 25, 2361–2371. 36368511 10.1016/j.jacc.2022.11.005

[4] Fuchs F. D. and Whelton P. K. , High Blood Pressure and Cardiovascular Disease, Hypertension. (2020) 75, no. 2, 285–292, 10.1161/hypertensionaha.119.14240. 31865786 PMC10243231

[5] Baffour P. K. , Jahangiry L. , Jain S. , Sen A. , and Aune D. , Blood Pressure, Hypertension, and the Risk of Heart Failure: a Systematic Review and meta-analysis of Cohort Studies, European Journal of Preventive Cardiology. (2024) 31, no. 5, 529–556, 10.1093/eurjpc/zwad344. 37939784

[6] Lee H. , Kwon S. H. , Jeon J. S. , Noh H. , Han D. C. , and Kim H. , Association Between Blood Pressure and the Risk of Chronic Kidney Disease in treatment-naïve Hypertensive Patients, Kidney Research and Clinical Practice. (2022) 41, no. 1, 31–42, 10.23876/j.krcp.21.099. 34974658 PMC8816410

[7] Weldegiorgis M. and Woodward M. , The Impact of Hypertension on Chronic Kidney Disease and end-stage Renal Disease is Greater in Men than Women: a Systematic Review and meta-analysis, BMC Nephrology. (2020) 21, no. 1, 10.1186/s12882-020-02151-7. PMC7687699 33238919

[8] Yang L. , Li J. , Wei W. et al., Blood Pressure Variability and the Progression of Chronic Kidney Disease: a Systematic Review and Meta-Analysis, Journal of General Internal Medicine. (2023) 38, no. 5, 1272–1281, 10.1007/s11606-022-08001-6. 36650323 PMC10110830

[9] Hezam A. A. M. , Shaghdar H. B. M. , and Chen L. , The Connection Between Hypertension and Diabetes and Their Role in Heart and Kidney Disease Development, Journal of Research in Medical Sciences. (2024) 29, no. 1, 10.4103/jrms.jrms_470_23. PMC11162087 38855561

[10] Petrie J. R. , Guzik T. J. , and Touyz R. M. , Diabetes, Hypertension, and Cardiovascular Disease: Clinical Insights and Vascular Mechanisms, Canadian Journal of Cardiology. (2018) 34, no. 5, 575–584, 10.1016/j.cjca.2017.12.005. 29459239 PMC5953551

[11] Nagata D. and Hishida E. , Elucidating the Complex Interplay Between Chronic Kidney Disease and Hypertension, Hypertension Research. (2024) 47, no. 12, 3409–3422, 10.1038/s41440-024-01937-8. 39415028

[12] Gallo G. and Savoia C. , Hypertension and Heart Failure: from Pathophysiology to Treatment, International Journal of Molecular Sciences. (2024) 25, no. 12, 10.3390/ijms25126661. PMC11203528 38928371

[13] Ndumele C. E. , Neeland I. J. , Tuttle K. R. et al., A Synopsis of the Evidence for the Science and Clinical Management of cardiovascular-kidney-metabolic (CKM) Syndrome: a Scientific Statement from the American Heart Association, Circulation. (2023) 148, no. 20, 1636–1664, 10.1161/cir.0000000000001186. 37807920

[14] Guideline for the Pharmacological Treatment of Hypertension in Adults, 2021, World Health Organization, Geneva. 34495610

[15] Mancia G. , Kreutz R. , Brunström M. et al., 2023 ESH Guidelines for the Management of Arterial Hypertension the Task Force for the Management of Arterial Hypertension of the European Society of Hypertension, Journal of Hypertension. (2023) 41, no. 12, 1874–2071, 10.1097/hjh.0000000000003480. 37345492

[16] McEvoy J. W. , McCarthy C. P. , Bruno R. M. et al., 2024 ESC Guidelines for the Management of Elevated Blood Pressure and Hypertension, European Heart Journal. (2024) 45, no. 38, 3912–4018, 10.1093/eurheartj/ehae178. 39210715

[17] Jones D. W. , Ferdinand K. C. , Taler S. J. et al., 2025 AHA/ACC/AANP/AAPA/ABC/ACCP/ACPM/AGS/AMA/ASPC/NMA/PCNA/SGIM Guideline for the Prevention, Detection, Evaluation, and Management of High Blood Pressure in Adults, Journal of the American College of Cardiology. (2025) 86, no. 18, 1567–1678, 10.1016/j.jacc.2025.05.007. 40815242

[18] Moran A. E. , Gupta R. , Pathni A. et al., Implementation of Global Hearts Hypertension Control Programs in 32 Low- and Middle-Income Countries, Journal of the American College of Cardiology. (2023) 82, no. 19, 1868–1884, 10.1016/j.jacc.2023.08.043. 37734459

[19] Schutte A. E. , Srinivasapura Venkateshmurthy N. , Mohan S. , and Prabhakaran D. , Hypertension in Low- and Middle-Income Countries, Circulation Research. (2021) 128, no. 7, 808–826, 10.1161/circresaha.120.318729. 33793340 PMC8091106

[20] Yusuf S. , Joseph P. , Rangarajan S. et al., Modifiable Risk Factors, Cardiovascular Disease, and Mortality in 155 722 Individuals from 21 high-income, middle-income, and low-income Countries (PURE): a Prospective Cohort Study, Lancet. (2020) 395, no. 10226, 795–808, 10.1016/s0140-6736(19)32008-2. 31492503 PMC8006904

[21] Mills K. T. , Stefanescu A. , and He J. , The Global Epidemiology of Hypertension, Nature Reviews Nephrology. (2020) 16, no. 4, 223–237, 10.1038/s41581-019-0244-2. 32024986 PMC7998524

[22] Ferrannini E. and Cushman W. C. , Diabetes and Hypertension: the Bad Companions, Lancet. (2012) 380, no. 9841, 601–610, 10.1016/s0140-6736(12)60987-8. 22883509

[23] Ncd Risk Factor Collaboration , Worldwide Trends in Hypertension Prevalence and Progress in Treatment and Control from 1990 to 2019: a Pooled Analysis of 1201 population-representative Studies with 104 Million Participants, Lancet. (2021) 398, no. 10304, 957–980. 34450083 10.1016/S0140-6736(21)01330-1 PMC8446938

[24] Mills K. T. , Bundy J. D. , Kelly T. N. et al., Global Disparities of Hypertension Prevalence and Control: a Systematic Analysis of Population-based Studies from 90 Countries, Circulation. (2016) 134, no. 6, 441–450, 10.1161/circulationaha.115.018912. 27502908 PMC4979614

[25] Zoccali C. , Mallamaci F. , Adamczak M. et al., Cardiovascular Complications in Chronic Kidney Disease: a Review from the European Renal and Cardiovascular Medicine Working Group of the European Renal Association, Cardiovascular Research. (2023) 119, no. 11, 2017–2032, 10.1093/cvr/cvad083. 37249051 PMC10478756

[26] Schiavone M. , Gobbi C. , Ratti A. , Ferrari G. , Villarini A. , and Meazza R. , Asymptomatic hypertension-mediated Organ Damage in Patients with or Without Moderate to Severe Chronic Kidney Disease, European Heart Journal. (2020) 41, no. Supplement_2, ehaa946–2715, 10.1093/ehjci/ehaa946.2715.

[27] Unger T. et al., 2020 International Society of Hypertension Global Hypertension Practice Guidelines, Hypertension. (2020) 75, no. 6, 1334–1357. 32370572 10.1161/HYPERTENSIONAHA.120.15026

[28] Whelton P. K. , Carey R. M. , Aronow W. S. et al., 2017 ACC/AHA/AAPA/ABC/ACPM/AGS/APhA/ASH/ASPC/NMA/PCNA Guideline for the Prevention, Detection, Evaluation, and Management of High Blood Pressure in Adults: a Report of the American College of Cardiology/American Heart Association Task Force on Clinical Practice Guidelines, Hypertension. (2018) 71, no. 6, e13–e115, 10.1161/hyp.0000000000000065. 29133356

[29] Williams B. , Mancia G. , Spiering W. et al., 2018 ESC/ESH Guidelines for the Management of Arterial Hypertension, European Heart Journal. (2018) 39, no. 33, 3021–3104, 10.1093/eurheartj/ehy339. 30165516

[30] Schmieder R. E. , Wassmann S. , Predel H. G. et al., Improved Persistence to Medication, Decreased Cardiovascular Events and Reduced All-Cause Mortality in Hypertensive Patients with Use of Single-Pill Combinations: Results from the START-Study, Hypertension. (2023) 80, no. 5, 1127–1135, 10.1161/hypertensionaha.122.20810. 36987918 PMC10112936

[31] Burnier M. and Egan B. M. , Adherence in Hypertension, Circulation Research. (2019) 124, no. 7, 1124–1140, 10.1161/circresaha.118.313220. 30920917

[32] Salam A. , Kanukula R. , Atkins E. et al., Efficacy and Safety of Dual Combination Therapy of Blood pressure-lowering Drugs as Initial Treatment for Hypertension: a Systematic Review and meta-analysis of Randomized Controlled Trials, Journal of Hypertension. (2019) 37, no. 9, 1768–1774, 10.1097/hjh.0000000000002096. 30986788

[33] King J. B. , An J. , Bellows B. K. et al., Single-Pill Combination Therapy for the Management of Hypertension: a Scientific Statement from the American Heart Association, Hypertension. (2026) 83, no. 3, 10.1161/hyp.0000000000000258. 41392889

[34] Coca A. , Whelton S. , Camafort M. , López-López J. , and Yang E. , Single-Pill Combination for Treatment of Hypertension: Just a Matter of Practicality or Is There a Real Clinical Benefit?, European Journal of Internal Medicine. (2024) 126, 16–25, 10.1016/j.ejim.2024.04.011. 38653633

[35] Rossignol P. , Massy Z. A. , Azizi M. et al., The Double Challenge of Resistant Hypertension and Chronic Kidney Disease, Lancet. (2015) 386, no. 10003, 1588–1598, 10.1016/s0140-6736(15)00418-3. 26530623

[36] Bouhlel I. , Clinical Profile and Predictors of Uncontrolled Arterial Hypertension in Hypertensive Obese Patients: Insights from the Tunisian Multicentric Observational Study NATURE HTN, Clinical Cardiovascular Research. (2025) 4, no. 10, 1–10, 10.58489/2836-5917/027.39825085

[37] Esmaeili F. , Karimi K. , Akbarpour S. et al., Patterns of General and Abdominal Obesity and Their Association with Hypertension Control in the Iranian Hypertensive Population: Insights from a Nationwide Study, BMC Public Health. (2025) 25, no. 1, 10.1186/s12889-024-21264-4. PMC11748872 39833748

[38] Parvanova A. , Reseghetti E. , Abbate M. , and Ruggenenti P. , *Mechanisms and Treatment of obesity-related Hypertension*—*Part 1: Mechanisms* , Clinical Kidney Journal. (2024) 17, no. 1, 10.1093/ckj/sfad282. PMC10768772 38186879

[39] Alsalloum M. A. , Albekery M. A. , and Alhomoud I. S. , Management of Hypertension in Chronic Kidney Disease: Current Perspectives and Therapeutic Strategies, Frontiers in Medicine. (2025) 12, 10.3389/fmed.2025.1630160. PMC12605421 41234923

